# A chain mediation model on COVID-19 symptoms and mental health outcomes in Americans, Asians and Europeans

**DOI:** 10.1038/s41598-021-85943-7

**Published:** 2021-03-19

**Authors:** Cuiyan Wang, Agata Chudzicka-Czupała, Michael L. Tee, María Inmaculada López Núñez, Connor Tripp, Mohammad A. Fardin, Hina A. Habib, Bach X. Tran, Katarzyna Adamus, Joseph Anlacan, Marta E. Aparicio García, Damian Grabowski, Shahzad Hussain, Men T. Hoang, Mateusz Hetnał, Xuan T. Le, Wenfang Ma, Hai Q. Pham, Patrick Wincy C. Reyes, Mahmoud Shirazi, Yilin Tan, Cherica A. Tee, Linkang Xu, Ziqi Xu, Giang T. Vu, Danqing Zhou, Natalie A. Chan, Vipat Kuruchittham, Roger S. McIntyre, Cyrus S. H. Ho, Roger Ho, Samuel F. Sears

**Affiliations:** 1 Institute of Cognitive Neuroscience, Faculty of Education, Huaibei Normal University, Huaibei, China; 2 Faculty of Psychology, SWPS University of Social Sciences and Humanities, Katowice, Poland; 3 University of the Philippines Manila, Manila, Philippines; 4 Department of Social, Work and Differential Psychology, Faculty of Psychology, Complutense University of Madrid, Somosaguas Campus, Madrid, Spain; 5 Department of Psychology, East Carolina University, Greenville, NC USA; 6 Department of Psychology, Zahedan Branch, Islamic Azad University, Zahedan, Iran; 7 Institute of Clinical Psychology, University of Karachi, Karachi, Pakistan; 8 Institute for Preventive Medicine and Public Health, Hanoi Medical University, Hanoi, 100000 Vietnam; 9 Bloomberg School of Public Health, Johns Hopkins University, Baltimore, MD 21205 USA; 10 DHQ Hospital Jhelum, Jhelum, Pakistan; 11 Institute for Global Health Innovations, Duy Tan University, Da Nang, Vietnam; 12 Institute for Preventive Medicine and Public Health, Hanoi Medical University, Hanoi, Vietnam; 13 Faculty of Medicine, Duy Tan University, Da Nang, Vietnam; 14 Department of Psychology, University of Sistan and Baluchestan, Zahedan, Iran; 15 Center of Excellence in Evidence-Based Medicine, Nguyen Tat Thanh University, Ho Chi Minh City, Vietnam; 16 Faculty of Medicine, Dentistry and Health, University of Sheffield, Sheffield, UK; 17 Southeast Asia One Health University Network (SEAOHUN), Chiang Mai, Thailand; 18 Mood Disorders Psychopharmacology Unit, University Health Network, University of Toronto, Toronto, Canada; 19 Department of Psychological Medicine, Yong Loo Lin School of Medicine, National University of Singapore, Singapore, Singapore; 20 Institute of Health Innovation and Technology (iHealthtech), National University of Singapore, Singapore, Singapore

**Keywords:** Diseases, Medical research, Risk factors

## Abstract

The novel Coronavirus-2019 (COVID-19) was declared a pandemic by the World Health Organization (WHO) in March 2020, impacting the lifestyles, economy, physical and mental health of individuals globally. This study aimed to test the model triggered by physical symptoms resembling COVID-19 infection, in which the need for health information and perceived impact of the pandemic mediated the path sequentially, leading to adverse mental health outcomes. A cross-sectional research design with chain mediation model involving 4612 participants from participating 8 countries selected via a respondent-driven sampling strategy was used. Participants completed online questionnaires on physical symptoms, the need for health information, the Impact of Event Scale-Revised (IES-R) questionnaire and Depression, Anxiety and Stress Scale (DASS-21). The results showed that Poland and the Philippines were the two countries with the highest levels of anxiety, depression and stress; conversely, Vietnam had the lowest mean scores in these areas. Chain mediation model showed the need for health information, and the perceived impact of the pandemic were sequential mediators between physical symptoms resembling COVID-19 infection (predictor) and consequent mental health status (outcome). Excessive and contradictory health information might increase the perceived impact of the pandemic. Rapid COVID-19 testing should be implemented to minimize the psychological burden associated with physical symptoms, whilst public mental health interventions could target adverse mental outcomes associated with the pandemic.

## Introduction

The coronavirus disease 2019 (COVID-19) was declared a pandemic by the World Health Organization (WHO) in March 2020. As of December 4 2020, the number of confirmed cases was 65,528,133, the number of death cases was 1,511,726 and the number of recovered cases was 45,371,073 worldwide^1^. While one may not necessarily contract COVID-19 during this time, certainly, his or her mental health is likely affected due to financial burden^2^, occupational injury due to potential risk of infection^3^ as well as the loss of livelihoods and opportunities^4^. The COVID-19 pandemic has become an urgent issue on global mental health and an unprecedented challenge for healthcare systems of all countries^5^. Emerging psychological disorders and mental health has been identified as the tenth leading research topic during the COVID-19 pandemic^6^.

A study of the early-stage COVID-19 pandemic in China found anxiety in 6.33% and depression in 17.17% of 600 respondents^7^. In other Asian countries, a new questionnaire, i.e., the Fear of COVID-19 Scale, was developed in Iran^8^, but this questionnaire did not measure other psychiatric symptoms such as depression. In Pakistan, mental illness poses a significant challenge to its under-resourced health care system^9^. In Italy, the healthcare system stretched to its limit because healthcare workers constituted10% of Italy’s confirmed COVID-19 cases^10^ As a result, recent guidelines recommended all healthcare workers should receive psychological support based on coping strategies for managing stress^11^. In Europe, the levels of psychiatric symptoms were generally low at the beginning of the COVID-19 pandemic. However, younger Spanish individuals with chronic diseases reported more symptoms than the rest of the population^12^. In the United States (US), Asian Americans were less likely to report psychiatric symptoms than Caucasian Americans during the COVID-19 pandemic^13^. Studies from China, Italy, Germany and Russia identified protective and risk person factors for mental health during COVID-19. Risk factors for adverse mental health include younger age^14^, especially young people who had to work outside their domicile^15^, reduced income^14^, family member infected by COVID-19^15^, having chronic diseases^14^, concerns related to COVID-19 infection for themselves or family members^14^, living alone^14^, having family conflicts^14^. Protective factors for mental health include disseminating reliable information^16^ and personal confidence by mastering knowledge of the pandemic^17^.

Researchers observed that mental health conditions such as depression and anxiety were affected by the pandemic^18^, but the underlying mechanisms remained unknown. Several behavioral theories could be applied to identify factors that influence mental health during the pandemic. One health behavior theory is the protection motivation theory developed by R.W. Rogers in 1975^19^. According to this theory, the COVID-19 pandemic might trigger the threat-appraisal and coping-appraisal processes^19^. The public would experience uncertainty and become very concerned about physical symptoms, which resemble COVID-19 infection. Due to the potential threat and impact of the pandemic, they would become worried that they did not have enough health information to protect themselves. For the coping-appraisal process, a person would search for health information to enhance understanding of the pandemic and take measures to reduce the risk of developing an infection^20^. The information-buffer hypothesis suggests that health information could buffer against physical symptom threats, thus reducing anxiety, depression, and stress. On the contrary, the overload of health information, especially inaccurate and misleading information, might lead to adverse mental health outcomes. Recently, Amanzio et al. (2020) proposed a theoretical framework to explain the association between health information, the psychological impact of a pandemic, physical symptoms and mental health outcomes based on the nocebo phenomenon for the infectious disease^21^. During the COVID-19 pandemic, conflicting and inaccurate health information (e.g., contradictory advice on face mask use) could lead to negative thinking and expectation^22^, resulting in the nocebo effect^21^, which ultimately lead to adverse mental health outcomes^23^. In summary, the physical symptoms resembling COVID-19 infection would trigger the need to search for health information, affecting the perceived impact of the pandemic and ultimately adverse mental health outcomes (i.e., anxiety, depression and stress).

**Figure 1:**
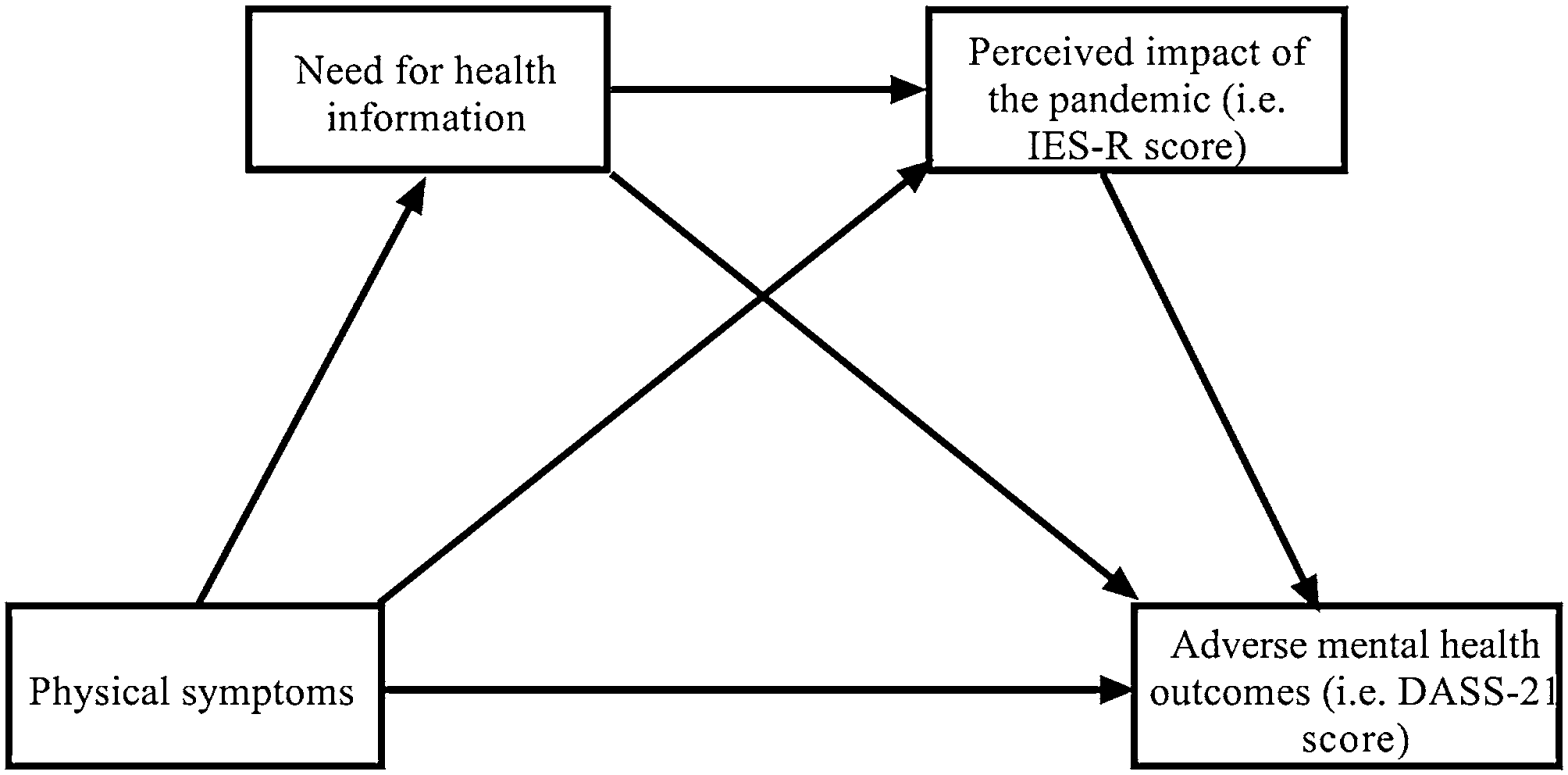
Proposed chain mediation model to explain the association between physical symptoms resembling COVID-19 infection and adverse mental health outcomes (i.e., anxiety, depression and stress).

To address the above research gaps, this study aimed to compare the mental health outcomes in the general population of 8 countries (China, Pakistan, Philippines, Iran, Poland, Spain, the US, Vietnam) during the COVID-19 pandemic. Based on existing theoretical perspectives, we constructed a chain mediation model to test the following hypothesis (see Fig. 1): (a) the physical symptoms resembling COVID-19 infection would be positively associated with adverse mental health outcomes (i.e., depression, anxiety and stress); (b) the need for health information would mediate the association between physical symptoms and adverse mental health outcomes; (c) the perceived impact of COVID-19 pandemic would mediate the association between physical symptoms and adverse mental health outcomes; (d) the need for health information and perceived impact of the pandemic would be sequential mediators in the association between physical symptoms and adverse mental health outcomes.

## Results

### Demographics of participants

There were 4612 participants from 8 countries (866 from China, 982 from Poland, 619 from the Philippines, 651 from Spain, 571 from the US, 391 from Iran, 419 from Pakistan, and 113 from Vietnam) who took part in the Global Mental Health Survey during the COVID-19 pandemic. Supplementary Table 1 compares the demographics of 8 countries. More than half of the participants were women in all countries (United States: 52.9% to 79.0% in Spain) except Pakistan. More than half of Chinese participants were below the age of 31 years. More than half of the Spanish participants were above the age of 41 years. The majority of Chinese, Iranian, Vietnamese and Polish respondents were married, while most Filipino respondents were single. More than 70% of participants have a university degree.

**Table 1:** Descriptive statistics and correlations of mean average score per item among subscales for all participants in 8 countries (N = 4612).

Sub-scales	*M* ± *SD*	1	2	3	4	5	6
1.Perceived psychological impact of COVID-19	2.24 ± 1.27	1					
2.DASS-21 Stress	1.75 ± 1.03	.539**	1				
3.DASS-21 Anxiety	1.90 ± 1.36	.539**	.735**	1			
4.DASS-21 Depression	1.78 ± 1.21	.485**	.767**	.723**	1		
5.Physical symptoms resembling COVID-19 infection	1.06 ± 1.43	.157**	.198**	.231**	.172**	1	
6.The need for health information	7.58 ± 3.42	.172**	-0.003	.055**	-0.001	.096**	1

^†^M refers to the mean average score per subscale. Mean average score = total score of subscale/number of items of a subscale.

*p < 0.05, **p < 0.01.

### Comparison of mental health outcomes among 8 countries

**Figure 2:**
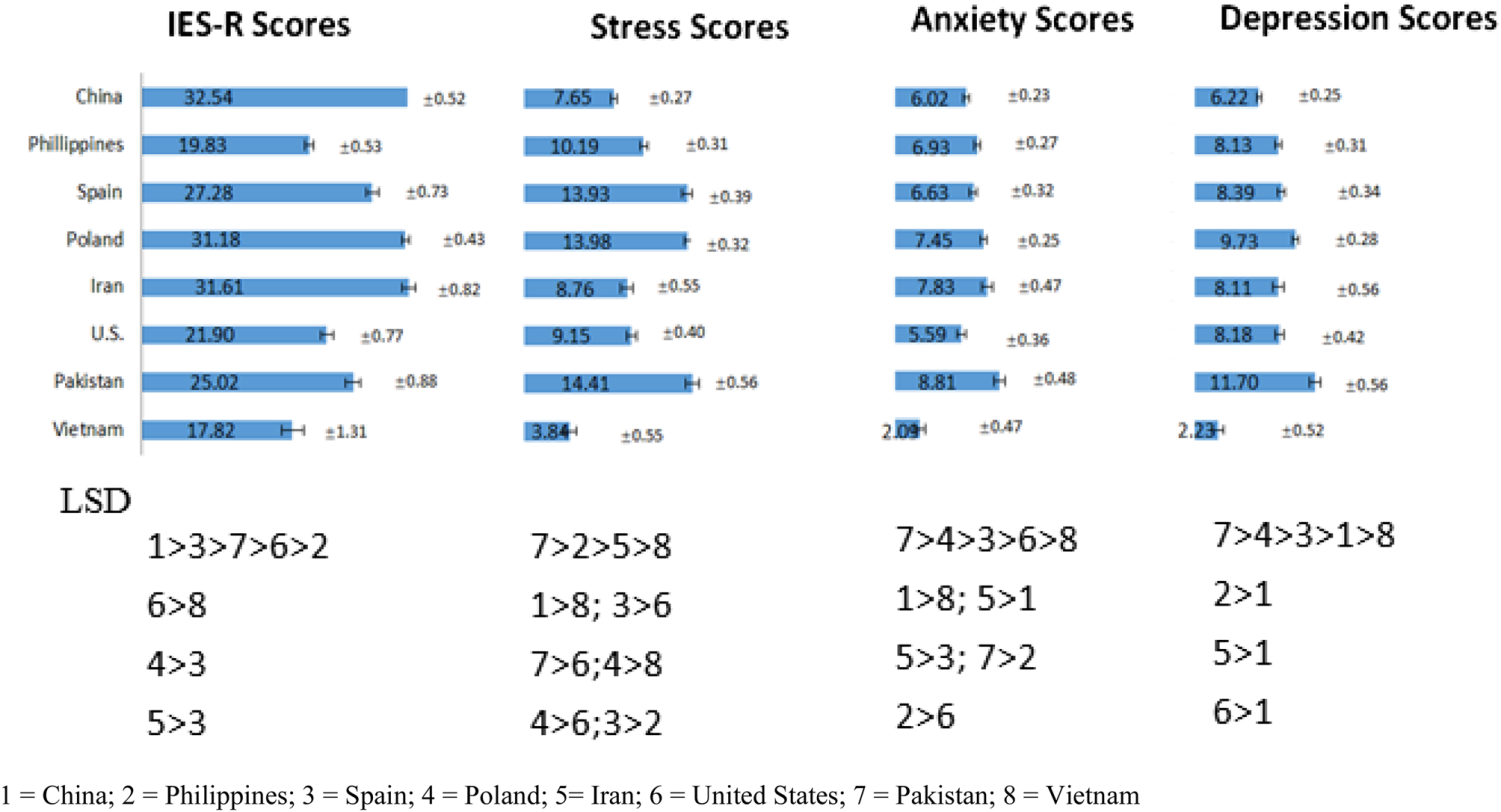
Comparison of Impact of Event Scale (Revised) IES-R and Depression, Anxiety and Stress Scale-21 (DASS-21) scores among 8 countries based on the least significant difference (LSD) analysis. 1 = China; 2 = Philippines; 3 = Spain; 4 = Poland; 5 = Iran; 6 = United States; 7 = Pakistan; 8 = Vietnam.

Figure 2 compares the IES-R and DASS-21 scores among all countries. China (mean = 32.54, SD = 0.52), Iran (mean = 31.61, SD = 0.82) and Poland (mean = 31.18, SD = 0.43) were the three countries with highest IES-R scores. There were significant differences in IES-R scores among 8 countries ( F(7, 4604) = 61.79, *η*^*2*^ = 0.086, p < 0.001). Pakistan (mean = 14.41, SD = 0.56), Poland (mean = 13.98, SD = 0.32) and Spain (mean = 13.93, SD = 0.39) were the three countries with highest DASS-21 stress scores. There were significant differences in DASS-21 stress scores among 8 countries (*F*(7, 4599) = 62.41, *η*^*2*^ = 0.087, *p* < 0.001). Pakistan (mean = 8.81, SD = 0.48), Iran (mean = 7.83, SD = 0.47) and Poland (mean = 7.45, SD = 0.25) were the three countries with highest DASS-21 anxiety scores. There were significant differences in DASS-21 anxiety scores among 8 countries (*F*(7, 4603 ) = 14.71, *η*^*2*^ = 0.022, *p* < 0.001). Pakistan (mean = 11.70, SD = 0.56), Poland (mean = 9.73, SD = 0.28) and Spain (mean = 8.39, SD = 0.34) were the three countries with highest DASS-21 depression scores. There were significant differences in DASS-21 depression scores among 8 countries (F(7, 4604) = 26.00, η^2^ = 0.038, p < 0.001). Vietnam has the lowest IES-R (mean = 17.82, SD = 1.31), stress (mean = 3.24, SD = 0.55), anxiety (mean = 2.09, SD = 0.47) and depression (mean = 2.23, SD = 0.52) scores. The LSD analysis showed that the scores of Vietnam were significantly lower than each of the other countries (p < 0.05).

### Physical symptoms resembling COVID-19 and need for health information

Supplementary Table 2 shows the frequency of physical symptoms that resemble COVID-19 infection. During the COVID-19 pandemic, the most common physical symptoms reported by the participants in 8 countries were headache (28.62%), cough (20.73%) and sore throat (19.7%). The least frequent physical symptoms were breathing difficulties (11.56%), rigors or chills (11.27%) and fever (10.99%). The prevalence of other physical symptoms was coryza (19.32%), myalgia (16.37%), dizziness (15.26%) and gastrointestinal symptoms (e.g., nausea, vomiting, diarrhea) (16.97%). Supplementary Table 3 shows the health information needs of participants from 8 countries. The top three information needs include understanding the effectiveness of drugs and vaccines available (70.22%), need for advice regarding treatment methods (64.36%) and information about local outbreaks (62.3%). Chinese participants reported the highest percentage for health information needs (> 90%).

**Table 2:** Results of mediation analysis.

Independent variables	Fit index			*B*	SE	*T*	95% CI	
	*R*	*R*^*2*^	*F*				LLCI	ULCI
**Dependent variable: need for health information**								
Constant	0.48	0.23	115.90***	9.70	0.12	80.88***	9.46	9.93
Physical symptoms				0.20	0.04	4.43***	0.11	0.28
**Dependent variable: perceived impact of the COVID-19 pandemic**								
Constant	0.34	0.12	47.36***	2.27	0.07	30.44***	2.12	2.41
Need for health information				0.03	0.01	5.62***	0.02	0.04
Physical symptoms				0.18	0.02	9.99***	0.14	0.21
**Dependent variable: DASS-21 anxiety score**								
Constant	0.60	0.36	180.77***	− 0.12	0.07	− 1.55	− 0.26	0.03
Need for health information				0.01	0.01	1.18	− 0.004	0.02
Perceived impact of the pandemic				0.59	0.01	44.22***	0.57	0.62
Physical symptoms				0.19	0.02	11.87***	0.16	0.22
**Dependent variable: DASS-21 depression score**								
Constant	0.55	0.31	144.37***	0.18	0.07	2.59**	0.04	0.31
Need for health information				-0.004	0.01	-0.80	-0.01	0.01
Perceived impact of the pandemic				0.49	0.01	39.11***	0.46	0.51
Physical symptoms				0.11	0.02	7.66***	0.09	0.14
**Dependent variable: DASS-21 stress score**								
Constant	0.62	0.39	209.76***	0.18	0.06	3.20**	0.07	0.28
Need for health information				-0.01	0.004	-1.31	-0.01	0.003
Perceived impact of the pandemic				0.46	0.01	45.89***	0.44	0.48
Physical symptoms				0.10	0.01	8.07***	0.07	0.12

*B* = unstandardized coefficient.

*** Significant at level p < 0.001.

**Table 3:** Results of the chain mediating effect based on Bootstrapping Test.

95% CI								
	Effect			SE		LLCI		ULCI
**Dependent variable: DASS-21 Anxiety scores**								
Physical symptoms → Need for health information → Anxiety								
	0.001			0.001		-0.001		0.004
Physical symptoms → Need for health information → Perceived impact of the pandemic → Anxiety								
	0.004			0.001		0.002		0.007
Physical symptoms → Perceived impact of the pandemic → Anxiety								
	0.105			0.01		0.08		0.13
**Dependent variable: DASS-21 Depression scores**								
Physical symptoms → Need for health information → Depression								
	-0.001			0.001		-0.003		0.001
Physical symptoms → Need for health information → perceived impact of the pandemic → Depression								
	0.003			0.001		0.001		0.006
Physical symptoms → Perceived impact of the pandemic → Depression								
	0.086			0.009		0.068		0.104
**Dependent variable: DASS-21 Stress scores**								
Physical symptoms → Need for health information → Stress								
	-0.001			0.001		-0.003		0.0003
Physical symptoms → Need for health information → Perceived impact of events → Stress								
	0.003			0.001		0.001		0.005
Physical health → Perceived impact of events → Stress								
	0.08			0.01		0.06		0.10

### Correlation of subscales

The mean average score per item for each subscale and correlations of sub-scales are displayed in Table 1. All the subscales were significantly correlated (p < 0.01) except for the need for health information with DASS-21 stress and depression subscales (p > 0.05). Physical symptoms resembling COVID-19 infection were positively and significantly associated with the perceived psychological impact of the pandemic as well as DASS-21 stress, anxiety and depression scores (p < 0.01). The need for health information was positively and significantly associated with the perceived psychological impact of the pandemic, DASS-21 anxiety score and physical symptoms (p < 0.01).

### The chain mediation model

Table 2 presents the results from the mediation of the need for health information and the perceived impact of the pandemic in the relationship between physical symptoms resembling COVID-19 infection and adverse mental health outcomes. In the first step, physical symptoms were found to have a significant and positive association with the need for health information (p < 0.001). In the second step, both physical symptoms and the need for health information were observed to show a significant and positive association with the perceived impact of the pandemic (p < 0.001). In the third step, mediation analysis was performed to assess the association between physical symptoms, the need for health information, the perceived impact of the pandemic and mental health outcomes. For anxiety, physical symptoms, the need for health information and the perceived impact of the pandemic were significantly and positively associated with anxiety (p < 0.001). For depression, physical symptoms, the need for health information and the perceived impact of the pandemic were significantly and positively associated with depression (p < 0.001). For stress, physical symptoms, the need for health information and the perceived impact of the pandemic were significantly and positively associated with stress (p < 0.001).

Table 3 shows the chain mediating effect of the need for health information and the perceived impact of the COVID-19 pandemic between physical symptoms and various mental health outcomes. For anxiety, the chain mediating effect of the need for health information and perceived impact of the COVID-19 pandemic between physical symptoms and anxiety was significant (effect = 0.004, 95% CI = 0.002–0.007). For depression, the chain mediating effect of the need for health information and perceived impact of the pandemic between physical symptoms and depression was significant (effect = 0.003, 95% CI = 0.001–0.006). For stress, the chain mediating effect of the need for health information and perceived impact of the pandemic between physical symptoms and depression was significant (effect = 0.003, 95% CI = 0.001–0.005).

**Figure 3:**
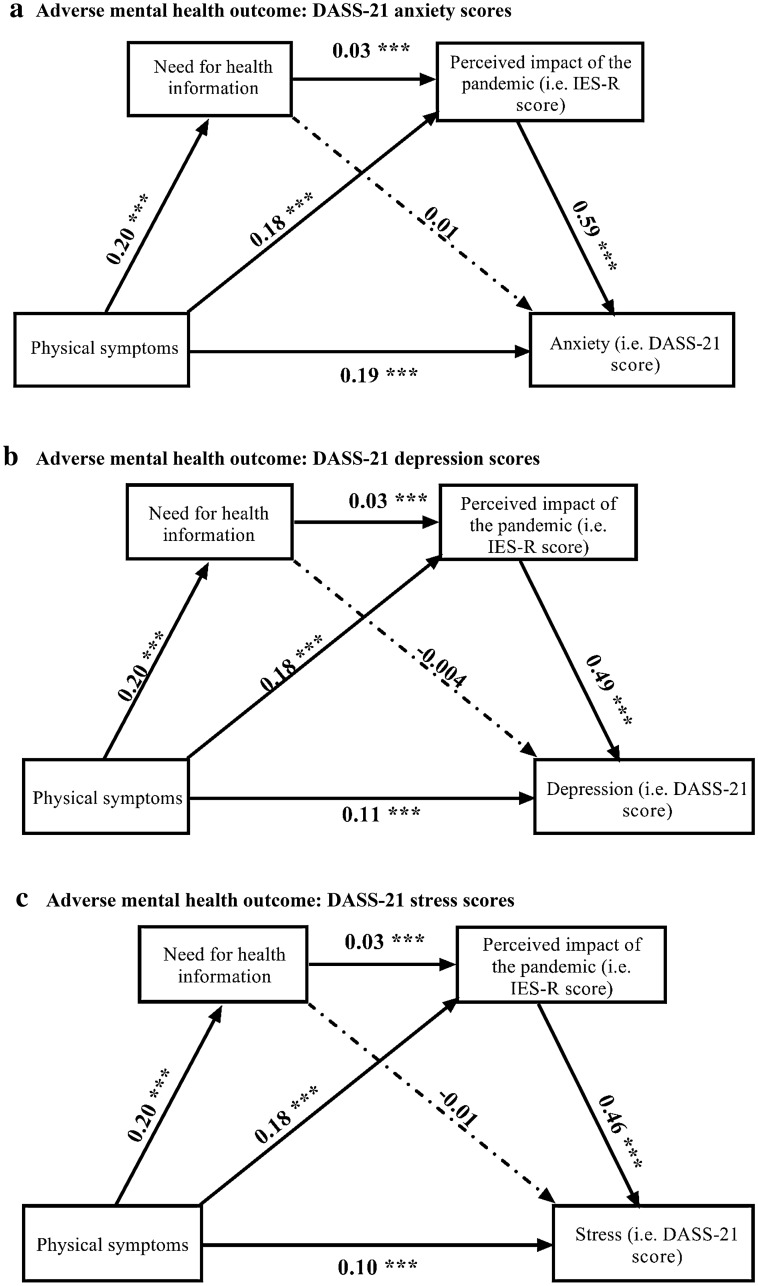
Tests of chain mediation model showed the indirect effect of need for health information, and the sequential indirect effects of the need for health information and perceived impact of the pandemic, in the association between physical symptoms resembling COVID-19 infection and adverse mental health outcomes. (**a) **Adverse mental health outcome: DASS-21 anxiety scores. *** Significant at level p < 0.001. (**b) **Adverse mental health outcome: DASS-21 depression scores. *** Significant at level p < 0.001. (**c)** Adverse mental health outcome: DASS-21 stress scores. *** Significant at level p < 0.001.

Figure 3a showed the chain mediating effect of the need for health information, and the sequential chain mediating effect for the need for health information and the perceived impact of the COVID pandemic in the association between physical symptoms and anxiety. All the paths in this model were significant (p < 0.001) except that the association between the need for health information and anxiety (B = 0.01, p > 0.05). Figure 3b showed the chain mediating effect of the need for health information, and the sequential chain mediating effect for the need for health information and the perceived impact of the COVID pandemic in the association between physical symptoms and depression. All the paths in this model were significant (p < 0.001) except that the association between the need for health information and depression (B = − 0.004, p > 0.05). Figure 3c showed the chain mediating effect of the need for health information, and the sequential chain mediating effect for the need for health information and the perceived impact of the COVID pandemic in the association between physical symptoms and stress. All the paths in this model were significant (p < 0.001) except that the association between the need for health information and stress (B = − 0.01, p > 0.05). For the three adverse mental health outcomes, the need for health information, when considered alone, did not act as a mediator.

## Discussion

The objective of this study was to compare the levels of DASS-21 scores and to rest the association between physical symptoms resembling COVID-19 infection and adverse mental health outcomes, as well as the mechanisms accountable for this association in multi-national populations across Asia, Europe and North America. The key findings were summarized as follows: (a) Poland and Pakistan were two countries with high levels of anxiety, depression and stress; (b) Vietnam had the lowest mean scores in anxiety, depression and stress; (c) Physical symptoms resembling COVID-19 infection was a risk factor for adverse mental health outcomes. Test of mediation showed that the need for health information explained partly of this mediation process. Although the need for health information did not act as a mediator when considered alone, there was a sequential mediating effect in which physical symptoms was associated with the need for health information, which in turn associated with higher perceived impact of the pandemic, which in turn associated with adverse mental health outcomes (i.e., anxiety, depression and stress).

Based on research conducted before the pandemic, the normative data for DASS-21 are as follows: the mean depression score was 3.87, the mean anxiety score was 2.95, and the mean stress score was 4.87^24^. In this study, the mean DASS-21 scores of all countries were higher than normative data except Vietnam. For IES-R, the means IES-R scores reported by healthy citizens after witnessing cardiopulmonary resuscitation (CPR) was 20.17.^25^. In this study, the mean IES-R scores of Chinese, Spanish, Polish, Iranian, American and Pakistani respondents were higher than healthy citizens witnessing CPR except for Filipino and Vietnamese. We observed that Pakistan and Poland were the two countries with the highest DASS-21 subscale scores. Before the COVID-19 pandemic, the World Happiness Report ranked the countries that had the greatest improvement of happiness from 2005–2008 to 2016–2018 as follows: Philippines (+ 0.860), Pakistan (+ 0.703), Poland (+ 0.445) and China (+ 0.426)^26^. In contrast, United States (− 0.446), Iran (− 0.713) and Spain (− 0.793) showed reduction in happiness scores from 2005–2008 to 2016–2018^26^. The COVID-19 pandemic might reverse the increase in happiness scores in Poland and Pakistan. Each country faced unique challenges during the pandemic. Respondents from Poland reported high DASS-21 stress, anxiety and depression scores. A recent Polish study found that loneliness was correlated with psychiatric symptoms and emotional response to physical health threat during the COVID-19 pandemic^27^. Furthermore, increased worry about the social isolation and concerns for financial problems was observed in lonelier Poles^27^. Additionally, the Polish media frequently reported that the healthcare system in Poland was not prepared to fight the pandemic, having to deal with staff shortages, deficit in medicine supplies and personal protective equipment (PPE) for health personnel or hospital closures, which may have had an impact on the mental health of the Poles^28^. Pakistani respondents reported high levels of DASS-21 stress, anxiety and depression scores, which may be related to perceptions of an incomplete response to COVID-19 due to poor sanitation, lack of basic preventive measures, lack of proper testing and medical facilities^28^. Pakistani health professionals started protesting and threatened to quit work due to a lack of PPE^28^. The unpreparedness and contradictory policies resulted in an alarmingly high COVID-19 spread and worsening mental health of Pakistani people, although data collected on mental health was during the peak time of the COVID-19 spread in the country. Chinese respondents reported the highest IES-R scores. China was the first country to report COVID-19, but the Chinese people were also accused of not being transparent about the COVID-19 and spreading the virus across the world^29^. The editor-in-chief of *The Lancet*, Richard Horton expressed concern about discrimination or revenge actions toward China and Chinese^30^. Iran ranked second in terms of high IES-R and DASS-21 anxiety scores. The economic sanctions that prevented medical supplies, equipment and drugs from arriving in Iran^31^ could lead to anxiety among Iranians during the pandemic.

In this study, Vietnamese respondents were found to have the lowest DASS-21 and IES-R scores. Coincidentally, news reports identified Vietnam as one of the best countries in adopting multiples effective measures that have been key to fighting the COVID-19 pandemic to date^32^. Effective measures include dissemination of health information^33^, engagement of grassroots healthcare system^34^ and village health collaborators^34^, as well as safeguarding the health of workforce^35^ to ensure minimal impact on the economy.

The current study is the first to demonstrate the mediation mechanism underlying the association between physical symptoms resembling COVID-19 infection and mental health outcomes. Based on the chain mediation model, physical symptoms were positively associated with higher anxiety, depression and stress. This result adds to previous studies that have suggested that real or perceived infection threats could lead to negative psychological reactions^36^, and people's anxieties were closely related to physical symptoms^37^. Experiencing physical symptoms that resemble COVID-19 infection could trigger hypochondriasis^38^, and a higher number of physical symptoms experienced could lead to strong disease conviction^39^. As a result, rapid diagnostic test development and implementation are crucial to alleviating adverse mental health outcomes when a person experiences physical symptoms similar to COVID-19 infection^40^. The uncertainty of potential threat during the early stage of the COVID-19 pandemic could trigger anxieties, depression and stress much more than fear^38^. The current study also identified the role of health information as a mediator in the link between physical symptoms and the perceived impact of the pandemic. During the strict lockdown, people were refrained from social interaction^41^ and spent more time at home and searching for health information online. Consistent with the protection motivation theory^19^, the need to search for more health information is triggered by activity in survival circuits that detects imminent threats of COVID-19. Nevertheless, the need for health information was not associated with adverse mental health outcomes, which might partially support the information-buffer hypothesis. The need for health information formed a sequential mediation path with the perceived impact of the pandemic on mental health outcomes. This finding is consistent with previous research about information-induced behavioral changes during the COVID-19 lockdowns^42^. Excessive health information might heighten the perceived impact of the pandemic through cyberchondria that is defined as the unfounded escalation of concerns about COVID-19 symptoms based on a review of search results and literature online^43^. According to the nocebo phenomenon^21^, conflicting health information (e.g., confused face mask policy)^22^, unproven conspiracy theories^44^ and rumors^45^ also enhanced the negative impact of the pandemic. In contrast, people who were likely to less frequently accessed health information were less anxious, depressed and stressed, and worried about the pandemic^46^. Our findings confirmed the second path of the indirect effect: that physical symptoms resembling COVID-19 infection was associated with a higher level perceived impact of the pandemic and led to adverse mental health outcomes. This finding is consistent with previous research that symptoms of emerging infectious diseases might lead to stigma and adverse mental health outcomes^47^. In summary, the current study provided evidence that the perceived impact of the COVID-19 pandemic was associated with the need for health information which was rooted in the physical symptoms resembling COVID-19 infection. Physical symptoms were associated with adverse mental health outcomes with sequential mediation by the need for health information and perceived impact of the pandemic.

The findings of this first multi-national study have several implications on public mental health strategies. Firstly, Kaslow et al. (2020) proposed that community mental health strategies include providing support groups, participating in health education outreach and disseminating mental wellness guides^48^. Furthermore, mental health professionals should offer online psychological interventions such as cognitive behavior therapy (CBT) and mindfulness-based therapy to improve the general population's mental health^49^. The COVID-19 pandemic provides an opportunity to introduce and promote telepsychiatry that overcomes the quarantine measures and geographical distance for mental health assistance^50^. Second, as physical symptoms resembling COVID-19 infection (e.g., headache, chills, breathing difficulty, dizziness, coryza) were associated with adverse mental health outcomes, the lack of testing for coronavirus could worsen mental health. There is an urgent need to develop accurate, rapid diagnostic tests in general practitioners' clinics, community and rural settings^51^. For low income countries, coronavirus testing should be easily accessible and free. A negative COVID-19 test result for members of the general population who present with physical symptoms may alleviate anxiety, depression and stress. Third, based on our findings, the WHO, governments and health authorities should provide regular updates on health information including effectiveness of prevention strategies, therapeutics, and vaccines and treatment methods. The study results could contribute reference information to various countries that need to monitor public mental health status and provide accurate and consistent health information during the pandemic^37^.

### Limitations

This study has several limitations. The first limitation was that the study population had different sociodemographic characteristics as compared to the world population. The respondent sampling method also compromised the representativeness of samples. The study population was female predominant (proportion of female in the study population: 68.55%; world population: 49.58%)^52^ and a high proportion of the study population possessed a university degree (proportion of degree holders in the study population: 70%; world population: 7%)^53^. The second limitation was sampling and selection bias because we could not reach out to potential respondents without Internet access in both countries. There was an uneven number of participants among 8 countries because 1938 Vietnamese participants were excluded due to incomplete questionnaires, and a smaller number of Iranian participants were recruited due to lack of Internet access in some areas of Iran. The third limitation was the cross-sectional nature of this study. Although the chain mediation model contributes to our understanding of the mediational factors that might influence the association between physical symptoms and adverse mental health outcomes, it cannot verify the temporal relationship. A longitudinal study is required to verify the direction of the paths further. The fourth limitation was that we did not record demographic data regarding the pre-existing mental illness of the study participants. The fifth limitation was that self-reported psychological impact levels, anxiety, depression and stress may not always be aligned with objective assessment by mental health professionals. Nevertheless, the perceived impact, anxiety, depression and stress are based on personal feelings, and self-reporting was paramount during the COVID-19 pandemic. The sixth limitation was that we could not confirm whether participants were seropositive to COVID-19 at the time of the survey because it was an online questionnaire-based study. Another possible limitation was the different recruitment periods of participants for each country and we planned to study the impact of COVID-19 during the peak periods that varied from country to country. Lastly, we were unable to calculate the response rate. For potential respondents who were not keen to participate in the online survey, no response was recorded, and we could not collect any information from them.

In conclusion, this multi-national study across three continents results provides empirical evidence that COVID-19 affected mental health worldwide. We found that Poland and Pakistan were two countries with the highest mean scores in IES-R and DASS-21 anxiety, depression and stress scales. In contrast, Vietnam had the lowest mean scores in IES-R and DASS-21 anxiety, depression and stress scales. The chain mediation model shows that the need for health information and the perceived impact of the pandemic exert sequential mediating effects on mental health outcomes in people who experience physical symptoms that resemble COVID-19 infection. It is hoped that these results will be public health values in formulating mental health strategies for the pandemic.

## Materials and methods

### Participants and questionnaires

The recruitment period for each country is listed as follows: China (February 28 to March 1, 2020), Philippines (March 28 to April 7, 2020), Spain (April 14 to 18, 2020), Iran (March 24 to 26, 2020), United States (April 21 to April 29, 2020), Pakistan (April 21 to July 6, 2020), Vietnam (April 7 to 14 2020) and Poland (March 22 to March 26, 2020). This study was approved by the institutional review boards of Complutense University of Madrid (Spain) (Protocol Number: IRB (Pr_2019_20_027), East Carolina University (The US) (Protocol Number: UMCIRB 20-000838), Hanoi Medical University (Vietnam) (Protocol Number: QD 75/QD-YHDP&YHDP), Huaibei Normal University (China) (Protocol Number: HBU-IRB-2020-002), Islamic Azad University (Iran) (Protocol Number: IRB-2020-001), University of Karachi (Pakistan) (Protocol Number: ICP-1 (101) 2698), the University of Philippines Manila (Protocol Number: UPMREB 2020-198-01) and the SWPS University of Social Sciences and Humanities (Poland) (Protocol Number: WKEB62/04/2020). This study was performed according to the Declaration of Helsinki, and the ethical principles in the Belmont Report. All participants were above the age of 18 years and provided informed consent prior to participation of this study.

This study used a theory-based questionnaire, the National University of Singapore (NUS) COVID-19 questionnaire, designed to examine the relationship between physical symptoms resembling COVID-19, health information required, the psychological impact of COVID-19 and mental health parameters. Its psychometric properties were established in the initial phase and peak of the COVID-19 epidemic^54,55^. The NUS COVID-19 questionnaire consisted of 3 subscales: (1) demographic data; (2) physical symptoms related to COVID-19 in the past 14 days, and (3) health information required for the COVID-19 pandemic. Demographic data about age, gender, education, household size, marital status, parental status and residential city in the past 14 days were collected. Physical symptoms related to COVID-19 included cough, fever, gastrointestinal and other symptoms. Respondents also rated their physical health status and stated their history of chronic medical illness. The health information required for the COVID-19 pandemic includes symptoms related to COVID-19, prevention and treatment advice, need for a regular update, knowledge in local transmission, the effectiveness of drugs and vaccines, travel advice, transmission methods and other countries' responses. The internal consistency of subscales on physical symptoms and the need for health information was examined using Cronbach alpha coefficients. Cronbach's alpha > 0.6 was considered acceptable reliability based on a previous theory-based questionnaire^56^. The Cronbach's alpha for physical symptoms and the need for health information subscales were 0.63 and 0.95, respectively.

The psychological impact of COVID-19 was measured using the Impact of Event Scale-Revised (IES-R). The IES-R is a self-administered questionnaire that has been well-validated in the American, European and Asian populations for determining the extent of psychological impact after exposure to a traumatic event (i.e., the COVID-19 pandemic) within one week of exposure^57–60^. This 22-item questionnaire is composed of three subscales, aiming to measure the mean avoidance, intrusion and hyperarousal^61^. The total IES-R score is divided into 0–23 (normal), 24 – 32 (mild psychological impact), 33–36 (moderate psychological impact) and > 37 (severe psychological impact)^62^. For the regression analysis, the cut-off score for high and low psychological impact was 24. In this study, the Cronbach's alpha for the different versions of IES-R are as follows: China: 0.949, Iran: 0.912, Pakistan: 0.95, Poland: 0.883, Philippines: 0.912, Spain: 0.948, the US: 0.959 and Vietnam: 0.92.

The mental health status of respondents was measured using the Depression, Anxiety and Stress Scale (DASS-21) and calculation of scores was based on a previous study^63^. DASS-21 has been used to assess mental health in American^64^, Asian^65,66^ and European^67^ populations. The internal consistency of DASS-21 stress, anxiety and depression scales was measured by the Cronbach's alpha. In this study, the Cronbach's alpha for different versions of DASS-21 is as follows: China: stress: 0.888, anxiety: 0.845, depression: 0.878; Iran: stress: 0.934, anxiety: 0.891, depression: 0.94; Pakistan: stress: 0.923, anxiety: 0.914, depression: 0.923; Philippines: stress: 0.839, anxiety: 0.784, depression: 0.889; Poland: stress: 0.890, anxiety: 0.854, depression: 0.886; Spain: stress: 0.895, anxiety: 0.876, depression: 0.89; The US: stress: 0.921, anxiety: 0.914, depression: 0.938 and Vietnam: stress: 0.864, anxiety: 0.866, depression: 0.904. For the regression analysis, the cut-off score for high stress score group was ≥ 35; the low stress score group was ≤ 10; high anxiety group was ≥ 20; low anxiety group was ≤ 6; high depression group was ≥ 28 and low depression group was ≤ 9. IES-R and DASS-21 were previously used in research related to the COVID-19 epidemic^54,58,68,69^..

### Statistical analysis

Descriptive statistics were calculated to compare demographic characteristics, physical symptoms and health service utilization, contact history, knowledge and concern, precautionary measure and additional health information variables among 8 countries. One-Way analysis of variance (ANOVA) compared the mean IES-R and DASS-21 scores between 8 countries to determine whether the associated population mean IES-R or DASS-21 scores were significantly different. If there were significant differences among 8 countries, the Least Significant Difference (LSD) would calculate the smallest significance between mean scores of two countries with different combinations. Any difference larger than the LSD is considered a significant result. We used Pearson's correlation to calculate the correlation coefficients between physical symptoms, the need for health information, and the perceived impact of COVID-19 pandemic and adverse mental health outcomes. Then we followed a stepwise method to construct the best fitting model for the mediated effects of the need for health information and the perceived impact of the pandemic. Mediation analyses were conducted by a regression-based macro for SPSS version 21.0^70^. In addition, a bootstrapping procedure with 2000 replications was run to test the chain mediation model. The significance levels of direct and indirect effects among the four factors (i.e., physical symptoms, health information requirement, the psychological impact of events and mental health parameters) and chain mediating effect would be determined. All tests were two-tailed, with a significance level of *p* < 0.05. Statistical analysis was performed on SPSS Statistic 21.0.

### Disclaimer

The findings and conclusions in this manuscript are those of the authors, and do not necessarily represent an official position of the affiliated institutions.

### Transparency declaration

The authors affirm that this manuscript is an honest, accurate, and transparent account of the study being reported; that no important aspects of the study have been omitted; and that any discrepancies from the study as planned (and, if relevant, registered) have been explained.

### Patient and Public Involvement statement

Patients or the public WERE NOT involved in the design, or conduct, or reporting, or dissemination plans of our research.

### Dissemination declaration

Dissemination to these groups is not possible/applicable.

## Supplementary Information

Supplementary Tables.

## Data Availability

The data that support the findings of this study are available on request from the corresponding author. The data are not publicly available due to privacy or ethical restrictions.
