# Supplementary material for: A chain mediation model on COVID-19 symptoms and mental health outcomes in Americans, Asians and Europeans
Source: Sci Rep. 2021 Mar 19;11:6481. doi: 10.1038/s41598-021-85943-7 (PMC7979938; doi:10.1038/s41598-021-85943-7)
Supplement: Supplementary file 1 — Supplementary Tables. [file 41598_2021_85943_MOESM1_ESM.docx]

 A Chain Mediation Model on COVID-19 Symptoms and Mental Health Outcomes in Americans, Asians and Europeans

Cuiyan Wang *PhD*^1^, Agata Chudzicka-Czupała *PhD^2^,* Michael Tee *MD^3^,* María Inmaculada López Núñez *PhD*^4^, Connor Tripp *MS^5^,* Mohammad A. Fardin *PhD^6^,* Hina A. Habib *PhD^7^*, Bach X. Tran *PhD*^8,9^, Katarzyna Adamus *PhD* ^2^, Joseph Anlacan *PhD* ^3^, Marta E. Aparicio García *PhD* ^4^, Damian Grabowsk *PhD* i^2^,Shahzad Hussain *PhD* ^10^, Men T. Hoang *PhD* ^11^, Mateusz Hetnał *PhD* ^2^, Xuan T. Le *MSc*^12^, Wenfang Ma *Msc*^1^, Hai Q. Pham *PhD* ^13^, Patrick Reyes *PhD* ^3^, Mahmoud Shirazi *PhD* ^14^, Yilin Tan *Msc*^1^*,* Cherica Tee *MD^3^*, Linkang Xu *Msc^1^,* Ziqi Xu *Msc^1^,* Giang T. Vu *PhD* ^15^, Danqing Zhou *MS^1^,* Natalie A. Chan *MBBS*^16^, Vipat Kuruchittham *PhD*^17^, Roger S. McIntyre *MD^18^,* Cyrus Ho *MBBS*^19^, *Roger Ho *MD^19,20^,* Samuel F. Sears *PhD* ^5^

1. Institute of Cognitive Neuroscience, Faculty of Education, Huaibei Normal University, Huaibei, China
2. Faculty of Psychology, SWPS University of Social Sciences and Humanities, Katowice, Poland
3. College of Medicine, University of the Philippines Manila
4. Dept. of Social, Work and Differential Psychology, Faculty of Psychology, Complutense University of Madrid, Somosaguas Campus, Madrid, Spain
5. East Carolina University, Department of Psychology, Greenville, NC, United States
6. Department of Psychology, Zahedan Branch, Islamic Azad University, Zahedan, Iran
7. Institute of Clinical Psychology, University of Karachi. Pakistan
8. Institute for Preventive Medicine and Public Health, Hanoi Medical University, Hanoi 100000, Vietnam.
9. Bloomberg School of Public Health, Johns Hopkins University, Baltimore, MD 21205, USA
10. DHQ Hospital Jhelum, Pakistan
11. Institute for Global Health Innovations, Duy Tan University, Da Nang, Vietnam.
12. Institute for Preventive Medicine and Public Health, Hanoi Medical University, Hanoi, Vietnam
13. Faculty of Medicine, Duy Tan University, Da Nang, Vietnam
14. Department of Psychology, University of Sistan and Baluchestan, Zahedan, Iran
15. Center of Excellence in Evidence-based Medicine, Nguyen Tat Thanh University, Ho Chi Minh City,Vietnam
16. Faculty of Medicine*,* Dentistry and Health, University of Sheffield, United Kingdom
17. Southeast Asia One Health University Network (SEAHOUN)
18. Mood Disorders Psychopharmacology Unit, University Health Network, University of Toronto, Toronto, Canada
19. Department of Psychological Medicine, Yong Loo Lin School of Medicine, National University of Singapore, Singapore
20. Institute of Health Innovation and Technology (iHealthtech), National University of Singapore, Singapore

 *Corresponding author: Dr Roger Ho, Department of Psychological Medicine, Yong Loo Lin School of Medicine, National University of Singapore

Email: pcmrhcm@nus.edu.sg

**Supplementary Table 1.** Comparison of demographics of the participants from eight countries.

| **Variable** | **China**  **(N=866)** | **Philippines**  **(N=619)** | **Spain**  **(N=651)** | **Iran**  **(N=391)** | **America**  **(N=571)** | **Pakistan**  **(N=419)** | **Vietnam**  **(N=** **113)** | **Poland**  **(N=982)** | **Total**  **N=4612** |
| --- | --- | --- | --- | --- | --- | --- | --- | --- | --- |
| ***Gender n* (%)** | | | | | | | | | |
| Male | 313(36.1) | 178(28.8) | 137(21.0) | 132(33.8) | 268(46.9) | 222(53.0) | 42(37.2) | 227(23.1) | 1519 |
| Female | 553(63.9) | 441(71.2) | 514(79.0) | 259(66.2) | 302(52.9) | 197(47.0) | 71(62.8) | 755(76.9) | 3092 |
| ***Age n* (%)** | | | | | | | | | |
| 18-30 | 643(74.2) | 226(36.5) | 92(14.1) | 151(38.6) | 140(24.5) | 170(40.6) | 23(20.4) | 217(22.1) | 1662 |
| 31-40 | 94(10.9) | 178(28.8) | 157(24.1) | 143(36.6) | 166(29.1) | 132(31.5) | 48(42.5) | 479(48.8) | 1397 |
| 41-49 | 90(10.4) | 129(20.8) | 196(30.1) | 65(16.6) | 111(19.4) | 72(17.2) | 21(18.6) | 216(22.0) | 900 |
| 50以上 | 39(4.5) | 86(13.9) | 206(31.6) | 32(8.2) | 154(27.0) | 45(10.7) | 20(17.7) | 70(7.1) | 652 |
| ***Education Level n* (%)** | | | | | | | | | |
| Secondary level and below | 56(6.5) | 2(0.3) | 13(2.0) | 12(3.1) | 4(0.7) | 4(1.0) | 1(0.9) | 16(1.6) | 108 |
| High School | 43(5.0) | 8(1.3) | 125(19.2) | **—** | 65(11.4) | 29(6.9) | 6(5.3) | 199(20.3) | 475 |
| Degree Holders | 767(88.6) | 609(98.4) | 513(78.8) | 379(96.9) | 500(87.6) | 386(92.1) | 106(93.8) | 767(78.1) | 4027 |
| ***Marital Status n* (%)** | | | | | | | | | |
| Single | 271(31.3) | 355(57.4) | 304(46.7) | 123(31.5) | 230(40.3) | 161(38.4) | 20(17.7) | 387(39.4) | 1851 |
| Married | 583(67.3) | 240(38.8) | 71(10.9) | 268(68.5) | 287(50.3) | 232(55.4) | 91(80.5) | 586(59.7) | 2358 |
| Divorced or Separated | 9(1.0) | 19(3.1) | 269(41.3) | **—** | 43(7.5) | 14(3.3) | 1(0.9) | N/A | 355 |
| Widowed | 3(0.3) | 5(0.8) | 7(1.1) | **—** | 11(1.9) | 12(2.9) | 1(0.9) | 9(0.9) | 48 |
| ***Parental Status n* (%)** | | | | | | | | | |
| Has children | 565(65.2) | 236(38.1) | 352(54.1) | 230(58.8) | 285(49.9) | 201(48.0) | 71(62.8) | N/A | 1940 |
| No Children | 301(34.8) | 383(61.9) | 299(45.9) | 161(41.2) | 208(36.4) | 218(52.0) | 42(37.2) | N/A | 1612 |
| ***Family Size n* (%)** | | | | | | | | | |
| 6 people and above | 124(14.3) | 187(30.2) | 7(1.1) | 40(10.2) | 18(3.2) | 168(40.1) | 13(11.5) | 45(4.6) | 602 |
| 3-5 people | 689(79.6) | 317(51.2) | 339(52.1) | 255(65.2) | 265(46.4) | 207(49.4) | 83(73.5) | 546(55.6) | 2701 |
| 2 people | 43(5.0) | 64(10.3) | 202(31.0) | 51(13.0) | 182(31.9) | 28(6.7) | 12(10.6) | 258(26.3) | 840 |
| 1 person | 10(1.2) | 51(8.2) | 103(15.8) | 45(11.5) | 106(18.6) | 16(3.8) | 5(4.4) | 133(13.5) | 469 |
| ***Employment Status n* (%)** | | | | | | | | | |
| Unemployed | 65(7.5) | 28(4.5) | 54(8.3) | 30(7.7) | 49(8.6) | 31(7.4) | 1(0.9) | 45(4.6) | 303 |
| Housewife | 24(2.8) | 26(4.2) | 4(0.6) | 41(10.5) | 18(3.2) | 51(12.2) | 1(0.9) | N/A | 165 |
| Retired | 6(0.7) | 10(1.6) | 29(4.5) | 12(3.1) | 42(7.4) | 8(1.9) | 4(3.5) | 28(2.9) | 139 |
| Student | 305(35.2) | 75(12.1) | 66(10.1) | 81(20.7) | 19(3.3) | 54(12.9) | 2(1.8) | 28(2.9) | 630 |
| Employed | 466(53.8) | 480(77.5) | 498(76.5) | 227(58.1) | 443(77.6) | 275(65.6) | 105(92.9) | 881(89.7) | 3375 |

N/A: not applicable

**Supplementary Table 2.** **Physical symptoms resembling COVID-19 infection reported by the participants from eight countries.**

| **Variable** | **China**  **(N=866)** | **Philippines**  **(N=619)** | **Spain**  **(N=651)** | **Iran**  **(N=391)** | **America**  **(N=571)** | **Pakistan**  **(N=419)** | **Vietnam**  **(N=** **113)** | **Poland**  **(N=982)** | **Total**  **N=4612** |
| --- | --- | --- | --- | --- | --- | --- | --- | --- | --- |
| ***Prolonged or Recurring Fever n* (%)** | | | | | | | | | |
| Yes | 5(0.6) | 5(0.8) | 13(2.0) | 367(93.9) | 6(1.1) | 73(17.4) | 0(0.0) | 38(3.9) | 507 |
| No | 861(99.4) | 614(99.2) | 638(98.0) | 24(6.1) | 565(98.9) | 346(82.6) | 113(100) | 944(96.1) | 4105 |
| ***Rigors or Chills n*(%)** | | | | | | | | | |
| Yes | 26(3.0) | 11(1.8) | 52(8.0) | 364(93.1) | 14(2.5) | 22(5.3) | 4(3.5) | 27(2.7) | 520 |
| No | 840(97.0) | 608(98.2) | 599(92.0) | 27(6.9) | 557(97.5) | 397(94.7) | 109(96.5) | 955(97.3) | 4092 |
| ***Headache n*(%)** | | | | | | | | | |
| Yes | 83(9.6) | 141(22.8) | 255(39.2) | 259(66.2) | 92(16.1) | 154(36.8) | 55(48.7) | N/A | 1039 |
| No | 783(90.4) | 478(77.2) | 396(60.8) | 132(33.8) | 479(83.9) | 265(63.2) | 58(51.3) | N/A | 2591 |
| ***Myalgia n*(%)** | | | | | | | | | |
| Yes | 63(7.3) | 79(12.8) | 156(24.0) | 330(84.4) | 7(1.2) | 42(10.0) | 12(10.6) | 66(6.7) | 755 |
| No | 803(92.7) | 540(87.2) | 495(76.0) | 61(15.6) | 564(98.8) | 377(90.0) | 101(89.4) | 916(93.3) | 3857 |
| ***Cough n(%)*** | | | | | | | | | |
| Yes | 125(14.4) | 90(14.5) | 80(12.3) | 301(77.0) | 54(9.5) | 129(30.8) | 27(23.9) | 150(15.3) | 956 |
| No | 741(85.6) | 529(85.5) | 571(87.7) | 90(23.0) | 517(90.5) | 290(69.2) | 86(76.1) | 832(84.7) | 3656 |
| ***Breathing Difficulties n*(%)** | | | | | | | | | |
| Yes | 5(0.6) | 47(7.6) | 28(4.3) | 352(90.0) | 18(3.2) | 43(10.3) | 5(4.4) | 35(3.6) | 533 |
| No | 861(99.4) | 572(92.4) | 623(95.7) | 39(10.0) | 553(96.8) | 376(89.7) | 108(95.6) | 947(96.4) | 4079 |
| ***Dizziness n*(%)** | | | | | | | | | |
| Yes | 56(6.5) | 25(4.0) | 48(7.4) | 352(90.0) | 16(2.8) | 36(8.6) | 21(18.6) | N/A | 554 |
| No | 810(93.5) | 594(96.0) | 603(92.6) | 39(10.0) | 555(97.2) | 383(91.4) | 92(81.4) | N/A | 3076 |
| ***Coryza n*(%)** | | | | | | | | | |
| Yes | 125(14.4) | 69(11.1) | 70(10.8) | 337(86.2) | 1(0.2) | 10(2.4) | 31(27.4) | 248(25.3) | 891 |
| No | 741(85.6) | 550(88.9) | 581(89.2) | 54(13.8) | 570(99.8) | 409(97.6) | 82(72.6) | 734(74.7) | 3721 |
| ***Sore Throat n*(%)** | | | | | | | | | |
| Yes | 96(11.1) | 90(14.5) | 89(13.7) | 304(77.7) | 40(7.0) | 82(19.6) | 20(17.7) | 186(18.9) | 907 |
| No | 770(88.9) | 529(85.5) | 562(86.3) | 87(22.3) | 531(93.0) | 337(80.4) | 93(82.3) | 796(81.1) | 3705 |
| ***Recurrent Fever with Cough or Breathing Difficulties n(%)*** | | | | | | | | | |
| Yes | 4(0.5) | 1(0.2) | 3(0.5) | — | 4(0.7) | 43(10.3) | 0(0.00) | N/A | 55 |
| No | 862(99.5) | 618(99.8) | 648(99.5) | — | 567(99.3) | 376(89.7) | 113(100) | N/A | 3184 |
| ***Nausea, Vomiting or Diarrhea n(%)*** | | | | | | | | | |
| Yes | — | 20(3.2) | 47(7.2) | 355(90.8) | 23(4.0) | 22(5.3) | 2(1.8) | N/A | 469 |
| No | — | 599(96.8) | 604(92.8) | 36(9.2) | 548(96.0) | 397(94.7) | 111(98.2) | N/A | 2295 |
| ***GP visit n*(%)** | | | | | | | | | |
| Yes | 33(3.8) | 24(3.9) | 67(10.3) | 43(11.0) | 44(7.7) | 127(30.3) | 6(5.3) | 235(23.9) | 579 |
| No | 833(96.2) | 595(96.1) | 584(89.7) | 348(89.0) | 527(92.3) | 292(69.7) | 80(70.8) | 747(76.1) | 4006 |
| ***Hospitalization n*(%)** | | | | | | | | | |
| Yes | 2(0.2) | 2(0.3) | 5(0.8) | 2(0.5) | 5(0.9) | 81(19.3) | — | 16(1.6) | 113 |
| No | 864(99.8) | 617(99.7) | 646(99.2) | 41(10.5) | 566(99.1) | 194(46.3) | 86(76.1) | 966(98.4) | 3980 |
| ***Tested for Covid-19 n(%)*** | | | | | | | | | |
| Yes | 8(0.9) | 4(0.6) | 18(2.8) | 7(1.8) | 16(2.8) | 82(19.6) | 2(1.8) | 235(23.9) | 372 |
| No | 858(99.1) | 615(99.4) | 633(97.2) | 36(9.2) | 555(97.2) | 188(44.9) | 84(74.3) | 747(76.1) | 3716 |
| ***Isolation n*(%)** | | | | | | | | | |
| Yes | 20(2.3) | 11(1.8) | 48(7.4) | 3(0.8) | 52(9.1) | 74(17.7) | 2(1.8) | 6(0.6) | 216 |
| No | 846(97.7) | 608(98.2) | 603(92.6) | 40(10.2) | 519(90.9) | 194(46.3) | 84(74.3) | 976(99.4) | 3870 |
| ***Health Assessment n*(%)** | | | | | | | | | |
| Not good or Very poor | 8(0.9) | 4(0.6) | 4(0.6) | 9(2.3) | 106(18.6) | 11(2.6) | 1(0.9) | 11(1.1) | 154 |
| Normal | 254(29.3) | 92(14.9) | 73(11.2) | 108(27.6) | 300(52.5) | 56(13.4) | 19(16.8) | 96(9.8) | 998 |
| Good or Very Good | 604(69.7) | 523(84.5) | 574(88.2) | 274(70.1) | 157(27.5) | 352(84.0) | 66(58.4) | 875(89.1) | 3425 |
| ***History of Chronic Disease n*(%)** | | | | | | | | | |
| Yes | 55(6.4) | 133(21.5) | 197(30.3) | 44(11.3) | 117(20.5) | 74(17.7) | 13(11.5) | 207(21.1) | 840 |
| No | 811(93.6) | 486(78.5) | 454(69.7) | 347(88.7) | 454(79.5) | 294(70.2) | 73(64.6) | 775(78.9) | 3694 |
| ***Health Insurance n*(%)** | | | | | | | | | |
| Yes | 799(92.3) | 346(55.9) | — | 41(10.5) | 490(85.8) | 133(31.7) | N/A | N/A | 1809 |
| No | 67(7.7) | 273(44.1) | — | 350(89.5) | 81(14.2) | 286(68.3) | N/A | N/A | 1057 |

N/A: not applicable

**Supplementary Table 3. Comparison of Health Information Needs about Covid-19 in the Participants of the Eight Countries.**

| **Variable** | **China**  **(N=866)** | **Philippines**  **(N=619)** | **Spain**  **(N=651)** | **Iran**  **(N=391)** | **America**  **(N=571)** | **Pakistan**  **(N=419)** | **Vietnam**  **(N=113)** | **Poland**  **(N=982)** | **Total**  **N=4612** |
| --- | --- | --- | --- | --- | --- | --- | --- | --- | --- |
| ***Understanding of symptoms related to Covid-19 n (%)*** | | | | | | | | | |
| Yes | 783(90.4) | 400(64.6) | 238(36.6) | 338(86.4) | 205(35.9) | 215(51.3) | 79(69.9) | N/A | 1475 |
| No | 83(9.6) | 219(35.4) | 413(63.4) | 53(13.6) | 366(64.1) | 204(48.7) | 34(30.1) | N/A | 1289 |
| ***Advice regarding prevention methods n (%)*** | | | | | | | | | |
| Yes | 804(92.8) | 408(65.9) | 363(55.8) | 328(83.9) | 195(34.2) | 251(59.9) | 76(67.3) | N/A | 1621 |
| No | 62(7.2) | 211(34.1) | 288(44.2) | 63(16.1) | 376(65.8) | 168(40.1) | 37(32.7) | N/A | 1143 |
| ***Advice regarding treatment methods n (%)*** | | | | | | | | | |
| Yes | 711(82.1) | 421(68.0) | 433(66.5) | 361(92.3) | 236(41.3) | 266(63.5) | 62(54.9) | N/A | 1779 |
| No | 155(17.9) | 198(32.0) | 218(33.5) | 30(7.7) | 335(58.7) | 153(36.5) | 51(45.1) | N/A | 985 |
| ***Need for regular information updates n (%)*** | | | | | | | | | |
| Yes | 802(92.6) | 416(67.2) | 340(52.2) | 352(90.0) | 277(48.5) | 229(54.7) | 88(77.9) | N/A | 1702 |
| No | 64(7.4) | 203(32.8) | 311(47.8) | 39(10.0) | 294(51.5) | 190(45.3) | 25(22.1) | N/A | 1062 |
| ***Understanding of local outbreaks n (%)*** | | | | | | | | | |
| Yes | 807(93.2) | 407(65.8) | — | 349(89.3) | 269(47.1) | 218(52.0) | 73(64.6) | N/A | 1316 |
| No | 59(6.8) | 212(34.2) | — | 42(10.7) | 302(52.9) | 200(47.7) | 40(35.4) | N/A | 796 |
| ***Need for more personalized information, such as providing advice for those with a past medical history n (%)*** | | | | | | | | | |
| Yes | 833(96.2) | 408(65.9) | 380(58.4) | 339(86.7) | 209(36.6) | 220(52.5) | 71(62.8) | N/A | 1627 |
| No | 33(3.8) | 211(34.1) | 271(41.6) | 52(13.3) | 362(63.4) | 196(46.8) | 42(37.2) | N/A | 1134 |
| ***Understanding of effectiveness of drugs or vaccines available n (%)*** | | | | | | | | | |
| Yes | 807(93.2) | 425(68.7) | 496(76.2) | 377(96.4) | 300(52.5) | 267(63.7) | 73(64.6) | N/A | 1938 |
| No | 59(6.8) | 194(31.3) | 155(23.8) | 14(3.6) | 271(47.5) | 148(35.3) | 40(35.4) | N/A | 822 |
| ***Understanding of number of infections and location n (%)*** | | | | | | | | | |
| Yes | 825(95.3) | 406(65.6) | 272(41.8) | 344(88.0) | 269(47.1) | 203(48.4) | 70(61.9) | N/A | 1564 |
| No | 41(4.7) | 213(34.4) | 379(58.2) | 47(12.0) | 302(52.9) | 212(50.6) | 43(38.1) | N/A | 1196 |
| ***Travel advice n (%)*** | | | | | | | | | |
| Yes | 834(96.3) | 372(60.1) | 288(44.2) | 283(72.4) | 189(33.1) | 188(44.9) | 43(38.1) | N/A | 1363 |
| No | 32(3.7) | 247(39.9) | 363(55.8) | 108(27.6) | 382(66.9) | 229(54.7) | 70(61.9) | N/A | 1399 |
| ***Understanding of modes of transmission of Covid-19 n (%)*** | | | | | | | | | |
| Yes | 815(94.1) | 396(64.0) | 337(51.8) | 352(90.0) | 203(35.6) | 220(52.5) | 71(62.8) | N/A | 1579 |
| No | 51(5.9) | 223(36.0) | 314(48.2) | 39(10.0) | 368(64.4) | 199(47.5) | 42(37.2) | N/A | 1185 |
| ***Understanding of what methods and strategies are being utilized by other countries n (%)*** | | | | | | | | | |
| Yes | 447(51.6) | 410(66.2) | 373(57.3) | 343(87.7) | 224(39.2) | 225(53.7) | 68(60.2) | N/A | 1643 |
| No | 419(48.4) | 209(33.8) | 278(42.7) | 48(12.3) | 347(60.8) | 194(46.3) | 45(39.8) | N/A | 1111 |

N/A: not applicable
